# Homoisoflavanones from *Agave tequilana* Weber

**DOI:** 10.3390/molecules15053295

**Published:** 2010-05-04

**Authors:** José Antonio Morales-Serna, Armando Jiménez, Rosa Estrada-Reyes, Carmen Marquez, Jorge Cárdenas, Manuel Salmón

**Affiliations:** Instituto de Química, Universidad Nacional Autónoma de México Circuito Exterior, Ciudad Universitaria, Coyoacán 04510, Mexico D.F., México; E-Mails: malonso@unam.mx (C.M.); rjcp@unam.mx (J.C.)

**Keywords:** phytochemistry, *Agavaceae*, *Agave tequilana*, homoisoflavanones, NMR

## Abstract

Three homoisoflavanones were isolated from the “piña” and leaves of *Agave tequilana* Weber. The compounds were identified as: 5,7-dihydroxy-3-(4-methoxybenzyl)-chroman-4-one (**1**), 7-hydroxy-3-(4-hydroxybenzyl)-chroman-4-one (**2**) and 4’-demethyl-3,9-dihydro-punctatin (**3**). This is the first phytochemical study carried out to *Agave tequilana* Weber.

## 1. Introduction

**Figure 1:**
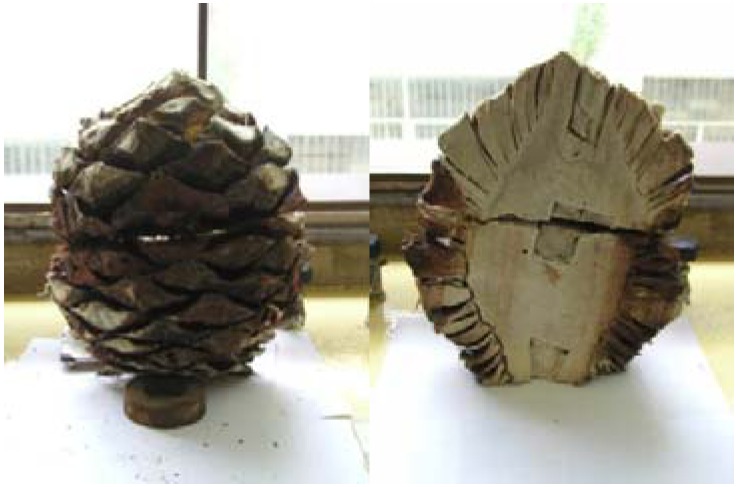
“Head” or “piña” (pine cone) from *Agave tequilana* Weber.

The Agavaceae is a family plant with nine genera and about 293 species. The most important genus is *Agave*, with about 166 species. Agaves are rosette, monocotyledoneous and monocarpic plants [1,2]. The agave plant has two main parts: the long spiked leaves from which sisal type fibres can be obtained and the “head” or “piña” from which juices are extracted for alcohol production. After the leaves are removed, the head looks like a pine cone, hence, the Spanish name piña (Figure 1). After removal of the leaves the piña weighs roughly 30–50 kg [3]. The most important diversity centre of agave is the Mexican territory, with species spread from southwestern United States through Central America, the Caribbean and into northern South America. Blue agave (*Agave tequilana* Weber), a member of the lily family, is grown extensively in the east and west regions of Guadalajara in Central Mexico. It is the raw material for production of the alcoholic beverage tequila. The origin-domination tequila name is applicable only to distillate from Weber blue agave grown within the limits of the State of Jalisco [4].

In an ample research work, Lopez *et al*. have described the carbohydrate composition [5,6,7] and volatile constituents [8] of *Agave tequilana* Weber. In this context and with the goal to contribute to enrich this knowledge we decided to perform a phytochemical study of *Agave tequilana* Weber. It is important to emphasize that to date no similar study has been reported in literature. Thus, this paper describes the isolation and structural elucidation of homoisoflavanones from ethyl acetate extracts of the “head” or “piña” and leaves.

## 2. Results and Discussion

The “piña” of *Agave tequilana* Weber (35 kg) was ground and extracted with water. The shredded agave fiber obtained from this first step was dried on air and at room temperature. Then, the solid was extracted with hexane and ethyl acetate at room temperature in a closed container several times. The extracts were concentrated under reduced pressure at 40 °C yielding 5 g and 13.5 g of dry extract, respectively. After removal of solvent, the hexane extract was fractionated on a silica gel column, eluted with a gradient hexane:ethyl acetate (9:1→1:9) yielding 9 fractions (IA-IXA). A sample of the first refined fraction (2 g) was analyzed by CG/MS to identify the compounds shown in Table 1.

**Table 1:** CG/MS analysis of a fraction of hexane extract from “piña” of *Agave tequilana* Weber.

Entry	Compound	Time	Area %
2	(*E*,*E*)-2,4-decadienal	10.38	0.98
3	Methyl 8-methyl-decanoate	18.45	0.89
4	Phenylmethyl benzoate	19.46	18.83
5	Palmitic acid	19.73	41.86
6	*Z*-9-Decanoic acid	21.97	18.36
7	5-Octadecine	22.24	11.60

**Figure 2:**
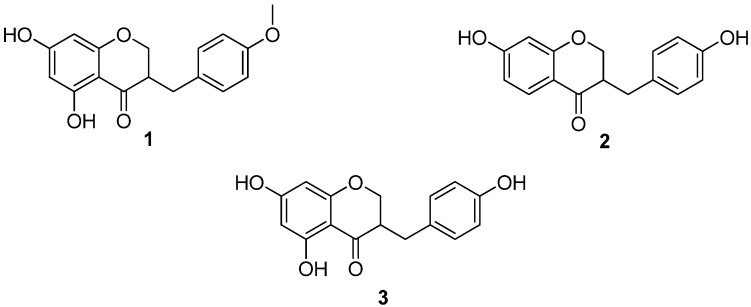
Homoisoflavanones from *Agave tequilana* Weber.

In the same way, the extract obtained with ethyl acetate was fractionated on a silica gel column, eluted with a gradient hexane:ethyl acetate (9:1→1:9) yielding 9 fractions (IB-IXB). From the first refined fraction we were able to isolate 120 mg of the homoisoflavanone **1** (Figure 2), which was identified by means of NMR and comparison with the data previously reported in the literature. 

The ^1^H-NMR spectrum of **1** was characterized by the presence of two sets of peaks from an ABX system attributable to the presence of non-equivalent protons at C-2 and C-9. These signals were observed at δ 4.0–4.3 and 2.6–2.8 ppm, respectively. The methine proton at C-3 gave resonance signals at about δ 2.81 (Table 2). The ^13^C-NMR spectrum also exhibited signals for C-2, C-3 and C-9 carbons at δ 68.9, 46.8 and 32.0 ppm, respectively (Table 3). The ^1^H-NMR spectrum of **1** showed an AA’BB’ system at δ 7.14 and 6.87 ppm (Table 2), due to the protons of ring B. The assignment was further confirmed by the ^13^C-NMR spectrum (δ 130 for C-2’/C-6’ and δ 114.1 for C-3’/C-5’, Table 3). Additionally, the ^1^H-NMR spectrum of **1** showed the presence of *meta* coupled signals at δ 5.99 and 6.92 ppm and these were assigned to H-6 and H-8 of ring A respectively (Table 2). These observations suggest that the C-5 and C-7 positions were oxygenated. Homoisoflavanone **1** is known to occur in *Eucomis bicolour* [9], *Ladebouria graminifolia* [10], *Ladebouria floribundia* [11] and *Agave barbadensis* [12]. The spectroscopic data given for that compound in these reports were identical to those measured by us in this study.

Additionally, the leaves of *Agave tequilana* Weber were dried on air and at room temperature and 650 g of them extracted several times with hexane, acetone and methanol at room temperature in a closed container. The extracts were concentrated under reduced pressure at 40 °C yielding 4.3 g, 3.9 and 44.2 g of dry extract, respectively. After purification on a chromatographic column sitosterol and stigmasterol were identified as the major components of the hexane extract, while glucose, sucrose and fructose were obtained from the methanolic extract. To our delight, the acetone extract resulted more interesting because was possible to isolate other homoisoflavanones. Thus, the acetone extract was fractionated on a silica gel column, eluting with a hexane-ethyl acetate (9:1→1:9) gradient yielding 9 fractions (IC-IXC). The homoisoflavanone **1** (5 mg) was isolated from the first refined fraction, while the homoisoflavanones **2** (3 mg) and **3 **(2.5 mg) were isolated from the third fraction (Figure 2). The ^1^H- NMR spectra of **2** show the presence of two ABX systems attributable to the presence of non-equivalent protons at C-2 and C-9. These signals were observed at δ 4.11–4.36 and 2.6–2.8 ppm, respectively. The methine proton at C-3 gave resonance signals at about δ 2.81 (Table 2). The ^13^C- NMR spectrum also exhibited signals for the C-2, C-3 and C-9 carbons at δ 68.9, 46.8 and 32.0 ppm, respectively (Table 3). The ^1^H-NMR spectrum of **2** showed an AA’BB’ system at δ 7.09 and 6.78 ppm (Table 2), due to the protons of ring B. The assignment was further confirmed by the ^13^C-NMR spectrum (δ 130 for C-2’/C-6’ and δ 114 for C-3’/C-5’, Table 3). For ring A, the ^1^H-NMR spectrum of **2** showed the presence of an *ortho* coupled signal at δ 7.83 that was assigned to H-5. In the same spectrum it is possible to see an ABX system at δ 6.52 which corresponds to H-6. Finally, the signal at δ 6.38 was assigned to H-8 (Table 2). The structure of this compound was confirmed by comparison with the data described in literature for the same homoisoflavanone isolated of the *Dracaena draco* [13,14] and *Dracaena cochinchinesis* [15].

**Table 2:** ^1^H-NMR spectral data, δ (*J* Hz) for compounds **1**, **2** and **3** in CDCl_3_ (300 MHz).

Proton	Homoisoflavanone		
	1	2	3
H-2b	4.11, *dd*, (11.7, 6.6)	4.15, *dd*, (11.4, 7.5)	4.14, *dd*, (11.4, 7.5)
H-3	2.81, *m*	2.8, *m*	2.8, *m*
H-5	-	7.83, *d*, (8.7)	-
H-6	5.99, *d*, (2.1)	6.52, *dd*, (8.7, 2.4)	6.0, *d*, (2.4)
H-8	5.92, *d*, (2.1)	6.38, *d*, (2.4)	5.98, *d*, (2.4)
H-9a	3.17, *dd*, (13.2, 3.6)	3.17, *dd*, (13.5, 4.2)	3.1, *dd*, (17.1, 4.2)
H-9b	2.73, *dd*, (13.2, 3.6)	2.65, *dd*, (13.5, 10.8)	2.65, *dd*, (17.1, 10.5)
H-2’	7.14, *d*, (8.4)	7.09, *d*, (8.4)	7.33, *d*, (8.7)
H-3’	6.87, *d*, (8.4)	6.78, *d*, (8.4)	6.88, *d*, (8.7)
H-5’	6.87, *d*, (8.4)	6.78, *d*, (8.4)	6.88, *d*, (8.7)
H-6’	7.14, *d*, (8.4)	7.09, *d*, (8.4)	7.33, *d*, (8.7)
4’-OMe	3.8, *s*	-	-

**Table 3:** ^13^C-NMR spectral data, δ (*J* Hz) for compounds **1**, **2** and **3** in CDCl_3_ (300 MHz).

Carbon	Homoisoflavanone		
	1	2	3
3	46.8	47.6	45.5
4	197.8	193	197.7
4a	102.5	103	101
5	164.5	128.8	163.6
6	96.6	114.5	95
7	164.5	167.9	166.7
8	95	103	94.7
8a	163.1	163.6	162.6
9	32	31.8	31.2
1’	129.7	130.2	128
2’	130	130.1	129.8
3’	114.1	114.1	115.1
4’	158.3	158.3	155.5
5’	114.1	114.1	115.1
6’	130	130.1	129.8
4’-OMe	55.3	-	-

In the case of homoisoflavanone 3, the ^1^H-NMR and ^13^C-NMR spectra are similar to the spectra of 1. The difference is the absence of the methoxyl substituent at C-4’, which shows signals at δ_H_ 3.8 and δ_C_ 55.3 ppm in the case of the 1. The ^1^H-NMR (Table 2) and ^13^C-NMR (Table 3) dates are identical to the described in the literature for the homoisoflavanone isolated from *Dracaena draco* [13], *Dracaena loureiri* [16,17], *Muscari comosum* [18], *Muscari neglectum* [19], *Chinodoxa luciliae* [20], *Ledeboria graminifolia* [10] and *Ledeboria floribunda* [11].

## 3. Experimental

### 3.1. General

The solvents used in this work were purchased from Aldrich. Flash column chromatography (FCC) was performed using flash silica gel (32–63 μm) and a solvent polarity correlated with TLC mobility was employed. The chromatographic columns were monitored by TLC carried out on 0.25 mm E. Merck silica gel plates. Developed TLC plates were visualized under a short-wave UV lamp and by heating plates that were dipped in ethanol/H_2_SO_4_ (15:1). Melting points, determined with Reichert apparatus, are uncorrected. Optical rotations were measured at 598 nm on a Jasco DIP-370 digital polarimeter using a 100 mm cell. NMR experiments were conducted on a Varian 300 MHz instrument using CDCl_3_ (99.9% D) as the solvent. Chemical shifts are reported in ppm with respect to TMS (tetramethylsilane).

### 3.2. Plant material

*Agave tequilana* Weber was obtained from the Jose Cuervo Tequila plant in Tequila Jalisco in Central Mexico in 2003. The leaves were cut off, keeping the stems and bases leaves, a part usually called “piña” due to it similarity to a pine cone fruit.

### 3.3. Extraction and Isolation

The fresh “piña” of *Agave tequilana* Weber (35 kg) were ground and extracted with water. The shredded agave fibrous obtained from this first step was dried on air and at room temperature for a week. Then, dry solid (3 kg) was extracted three times, at room temperature for 48 h with hexane (12 L) in a closed container. The combined extracts were concentrated under reduced pressure at 40 °C to give a viscous concentrate (5 g). In a second stage, the solid residue was extracted with EtOAc (12 L) three times for 48 h, at room temperature in a closed container. After solvent elimination 13.5 g of a viscous concentrate was obtained.

The hexane extract was fractionated on a silica gel column, eluted with a hexane-ethyl acetate gradient (9:1→1:9) yielding 9 fractions (IA-IXA). The quantitative determination of the volatile compounds hexanal, (*E*,*E*)-2,4-decadienal, methyl 8-methyldecanoate, phenylmethyl benzoate, palmitic acid, *Z*-9-decanoic acid and 5-octadecene from fraction IIA, was carried out using gas chromatography with flame ionization detection, as reported in the literature [21].

The residue obtained with AcOEt (13.5 g) was dissolved in CH_2_Cl_2_, Celite was added, and after the solvent had been removed *in vacuo*, the extract was subjected to silica gel chromatography eluting with a hexane-ethyl acetate gradient (9:1→1:9) to give 9 fractions (IB-IXB). The homoisoflavanone **1 **(120 mg) was obtained from fraction IIB by flash chromatography using hexane-ethyl acetate as eluent (8:2).

The air dried powdered leaves of *Agave tequilana* Weber (650 g) were extracted three times with hexane, acetone and methanol (3 L), at room temperature for 48 h, to give 4.3 g, 3.9 and 44.2 g of dry extract, respectively. Sitosterol and stigmasterol were obtained from the hexane extract, and glucose, sucrose and fructose were identified from methanolic extract, using gas chromatography with flame ionization detection.

The acetone extract was dissolved in CH_2_Cl_2_, Celite was added, and after the solvent had been removed *in vacuo*, the extract was subjected to silica gel chromatography eluting with a hexane-ethyl acetate gradient (9:1→1:9) to give 9 fractions (IC-IXC). The homoisoflavanone **1** (5 mg) was obtained from fraction IC. From fraction IIIC were isolated the homoisoflavanones **2** (3 mg) and **3 **(2.5 mg).

*5,7-Dihydroxy-3-(4-methoxybenzyl)-chroman-4-one* (**1**). IR-KBr Pellet (ν, cm^-1^): 3384, 2922, 2851, 1639, 1454, 1379, 1270, 1161, 833; ^1^H-NMR and ^13^C-NMR, see Table 1 and Table 2; HRMS: [M]^+^*m/z*: 300.0992. Calc. for C_17_H_16_O_5_ 300.0998.

*7-Hydroxy-3-(4-hydroxybenzyl)-chroman-4-one* (**2**): IR- KBr Pellet (ν, cm^-1^): 3415, 2852, 1646, 1514, 1459, 1245, 1163, 1019; ^1^H-NMR and ^13^C-NMR, see Table 1 and Table 2; HRMS: [M]^+^*m/z*: 270.0887. Calc. for C_16_H_14_O_4_ 270.0892.

*4’-Demethyl-3,9-dihydropunctatin* (**3**): IR- KBr Pellet (ν, cm^-1^): 3384, 2920, 2851, 1647, 1514, 1456, 1376, 1241, 1132, 850; ^1^H-NMR and ^13^C-NMR, see Table 1 and Table 2; HRMS: [M]^+^*m/z*: 286.0835. Calc. for C_16_H_14_O_5_ 286.0841

## 4. Conclusions

In summary, we were able to isolate three known compounds from the EtOAc and acetone extracts of *Agave tequilana* Weber. These compounds belong to a small homogeneous group of naturally occurring oxygen heterocycles called homoisoflavanones, whose basic structure consists of a 16 carbon skeleton, including a chromanone system with a benzyl group at position 3. It is the first time that the compounds **2** and **3** have been isolated from the genus *Agave*. Finally, is important to remark that, the value of present study lies in the importance of the plant to the tequila industry.
